# Association of Sociodemographic Factors and Blood Group Type With Risk of COVID-19 in a US Population

**DOI:** 10.1001/jamanetworkopen.2021.7429

**Published:** 2021-04-05

**Authors:** Jeffrey L. Anderson, Heidi T. May, Stacey Knight, Tami L. Bair, Joseph B. Muhlestein, Kirk U. Knowlton, Benjamin D. Horne

**Affiliations:** 1 Intermountain Medical Center Heart Institute, Salt Lake City, Utah; 2 University of Utah School of Medicine, Salt Lake City; 3 Stanford University, Stanford, California

## Abstract

This case-control study examined the association of sociodemographic factors and blood group type with risk of SARS-CoV-2 infection and severity of COVID-19.

## Introduction

The observed variability in susceptibility to SARS-CoV-2 and severity of the ensuing COVID-19 have raised intense interest in their environmental and genetic risk factors. An early report from China^1^ suggested that blood group A was associated with increased susceptibility and blood group O was associated with reduced susceptibility to SARS-CoV-2 infection. These reports motivated widespread interest in examining ABO blood groups as potential COVID-19 risk factors. Subsequent studies from Italy and Spain^2^ reported that blood group A was associated with an increased risk of severe COVID-19 and blood group O was associated with a reduced risk. In contrast, a large Danish study^3^ implicated disease susceptibility but not severity. However, observations from Boston, Massachussets,^4^ and New York, New York,^5^ did not confirm any specific associations between ABO blood group and disease. The controversy raised by these contrasting reports led to this case-control study.

## Methods

Our objective in this case-control study was to independently test whether blood type is associated with SARS-CoV-2 susceptibility and COVID-19 severity. The study was approved by the Intermountain Medical Center institutional review board with a waiver of consent because the study represented no more than minimal privacy risk to individuals. This study is reported following the Strengthening the Reporting of Observational Studies in Epidemiology (STROBE) reporting guideline.

Intermountain Healthcare, a nonprofit, integrated health care system of 24 hospitals and 215 clinics in Utah, Idaho, and Nevada, generated a SARS-CoV-2 and COVID-19–specific electronic health records database. We searched this database for individuals who were tested for SARS-CoV-2 between March 3 and November 2, 2020, and had a recorded blood type. For individuals who underwent multiple tests, the first test with a positive result was chosen, otherwise the first test with a negative result was used. We compared positive vs negative test results, hospitalized vs nonhospitalized patients, and intensive care unit (ICU) vs non-ICU patients. Infectivity was determined by SARS-CoV-2–specific polymerase chain reaction testing of nasal swabs or saliva samples. Analysis of variance assessed associations across ABO groups. Odds ratios (ORs) between ABO groups were assessed by logistic regression adjusted for age, sex, and Rh factor. *P* values were 2-sided, and statistical significance was set at *P* < .167 for each of the 3 primary comparisons. Statistical significance was set at *P* = .006 for assessment of ORs because each set underwent 9 comparisons. Data were analyzed from November 20, 2020, to February 26, 2021.

## Results

A total of 107 796 individuals (mean [SD] age, 42.0 [17.8] years; 82 875 [76.9%] women) who had been tested for SARS-CoV-2 infection were included in the study. Further demographic characteristics of the studied population and ABO blood group associations are shown in Table 1. Among individuals with COVID-19, hospitalization was associated with male sex (1165 men [50.1%] hospitalized vs 1871 men [20.5%] not hospitalized) and age (mean [SD] age among hospitalized patients, 57.0 [18.1] years vs 41.4 [14.9] years among nonhospitalized individuals). Admission to an ICU was also associated with male sex (426 men [61.8%] admitted to ICUs vs 725 men [44.8%] not admitted) and age (mean [SD] age among patients admitted to ICUs, 60.7 [158] years vs 55.4 [18.8] years in patients not admitted). Non-White race, which included African American, American Indian or Alaskan Native, Native Hawaiian or Pacific Islander, Asian, and unknown or declined to answer, was associated with viral positivity (1592 individuals [13.9%] with positive results vs 6610 individuals [6.9%] with negative results) and hospitalization (556 patients [23.9%] hospitalized vs 1036 individuals [11.3%] not hospitalized). Blood type was not associated with disease susceptibility or severity, including viral positivity, hospitalization, or ICU admission (Table 1). Compared with type O blood, type A was not associated with increased viral positivity (OR, 0.97 [95% CI, 0.93-1.01]; *P* = .11), hospitalization (OR, 0.89 [95% CI, 0.80-0.99]; *P* = .03), or ICU admission (OR, 0.84 [95% CI, 0.69-1.02]; *P* = .08) (Table 2). Similarly, types B and AB were not associated with worse outcomes than type O. Analyses restricted to White race produced similar results (Table 2).

**Table 1.** Individuals’ Characteristics by SARS-CoV-2 Positivity, Hospitalization, and ICU Admission

Characteristic	Individuals, No. (%)		*P* value
**Tested for SARS-CoV-2**			
	Negative results	Positive results	
No.	96 328	11 468	
Age, mean (SD), y	45.1 (17.9)	44.6 (16.8)	.003
Sex			
Women	74 439 (77.3)	8436 (73.6)	<.001
Men	21 889 (22.7)	3032 (26.4)	
White race^a^	89 718 (93.1)	9876 (86.1)	<.001
Blood type^b^			
A	38 872 (40.4)	4545 (39.6)	.24
B	8912 (9.3)	1037 (9.0)	
AB	3145 (3.3)	365 (3.2)	
O	45 399 (47.1)	5521 (48.1)	
**With positive SARS-CoV-2 results, by hospitalization status**			
	Nonhospitalized	Hospitalized	
No.	9142	2326	
Age, mean (SD), y	41.4 (14.9)	57.0 (18.1)	<.001
Sex			
Women	7271 (79.5)	1165 (50.1)	<.001
Men	1871 (20.5)	1161 (49.9)	
White race^a^	8106 (88.7)	1770 (76.1)	<.001
Blood type^b^			
A	3648 (39.9)	897 (38.6)	.54
B	832 (9.1)	205 (8.8)	
AB	286 (3.1)	79 (3.4)	
O	4376 (47.9)	1145 (49.2)	
**Hospitalized, by ICU status**			
	Non-ICU	ICU	
No.	1620	706	
Age, mean (SD), y	55.4 (18.8)	60.7 (15.8)	<.001
Sex			
Women	895 (55.2)	270 (38.2)	<.001
Men	725 (44.8)	436 (61.8)	
White race^a^	1264 (78.0)	506 (71.7)	.002
Blood type^b^			
A	640 (39.5)	257 (36.4)	.27
B	144 (8.9)	61 (8.6)	
AB	59 (3.6)	20 (2.8)	
O	777 (48.0)	368 (52.1)	

Abbreviation: ICU, intensive care unit.

^a^ Race was self-defined. Other races included African American (865 individuals [0.8%]); American Indian or Alaskan Native (788 individuals [0.7%]), Native Hawaiian or Pacific Islander (1661 individuals [1.5%]), Asian (1275 individuals [1.2%]), and unknown or declined to answer (3663 individuals [3.4%]).

^b^ ABO typing generally preceded or was concurrent with polymerase chain reaction testing (101 040 individuals [93.7%]). Significance across ABO groups for each of the 3 end points was set at *P* < .0167.

**Table 2.** Risk of Positive SARS-CoV-2 Test Results, Hospitalization Among Individuals With Positive SARS-CoV-2 Test Results, and ICU Admission Among Patients Hospitalized With COVID-19 by Blood Type^a^

Outcome	Type A vs Type O, OR (95% CI)	*P* value	Type B vs Type O, OR (95% CI)	*P* value	Type AB vs Type O, OR (95% CI)	*P* value	
All individuals							
Positive test results	0.97 (0.93-1.01)	.11	0.96 (0.89-1.03)	.25	0.96 (0.86-1.07)	.48	
Hospitalized	0.89 (0.80-0.99)	.03	0.91 (0.75-1.09)	.30	1.02 (0.77-1.35)	.91	
ICU admission	0.84 (0.69-1.02)	.08	0.89 (0.64-1.23)	.47	0.69 (0.40-1.18)	.17	
White individuals only							
Positive test results	0.97 (0.93-1.01)	.17	0.94 (0.87-1.01)	.10	0.94 (0.83-1.07)	.34	
Hospitalized	0.88 (0.78-0.99)	.04	0.92 (0.74-1.13)	.42	0.92 (0.66-1.28)	.63	
ICU admission	0.89 (0.71-1.11)	.30	0.94 (0.64-1.39)	.76	0.81 (0.43-1.53)	.51	

Abbreviations: ICU, intensive care unit; OR, odds ratio.

^a^ Models were adjusted for age, sex, and Rh factor. Given that each set underwent 9 comparisons, significance for individual comparisons was set at *P* = .006.

## Discussion

With contrasting reports from China,^1^ Europe,^2,3^ Boston,^4^ New York,^5^ and elsewhere,^6^ we embarked on a large, prospective case-control study that included more than 11 000 individuals who were newly infected with SARS-CoV-2, and we found no ABO associations with either disease susceptibility or severity. The smaller sample sizes and retrospective, observational nature of many prior studies, in addition to their striking heterogeneity of ABO associations with disease susceptibility and severity, could be due to chance variations, publication bias, differences in genetic background, geography and environment, and viral strains. The ABO gene is highly polymorphic, and ABO blood groups are distributed differently across ancestries and geographies. Differing disease susceptibilities could be due to natural antibodies or prothrombotic effects with nongroup O.^6^ The Utah population is dominated by Northern European ancestry, with a minority contribution of Hispanic/Latinx and other ethnicities. However, our results are similar among individuals of White race alone.

Given the large and prospective nature of our study and its strongly null results, we believe that important associations of SARS-CoV-2 and COVID-19 with ABO groups are unlikely and will not be useful factors associated with disease susceptibility or severity on either an individual or population level for similar environments and ancestries. Additional studies, closely controlled for genetics, geography, and viral strain, are required before accepting blood group as a determinant of predisposition to or severity of COVID-19.
